# Discovery of a Novel Bat Gammaherpesvirus

**DOI:** 10.1128/mSphere.00016-16

**Published:** 2016-02-17

**Authors:** Kurtis M. Host, Blossom Damania

**Affiliations:** a Lineberger Comprehensive Cancer Center, University of North Carolina at Chapel Hill, Chapel Hill, North Carolina, USA; b Department of Microbiology and Immunology, University of North Carolina at Chapel Hill, Chapel Hill, North Carolina, USA

**Keywords:** *Myotis velifer incautus*, bat, BGHV8, gammaherpesvirus, *Myotis* gammaherpesvirus 8

## Abstract

Zoonosis is the leading cause of emerging infectious diseases. In a recent article, R. S. Shabman et al. (mSphere 1[1]:e00070-15, 2016, http://dx.doi.org/10.1128/mSphere.00070-15) report the identification of a novel gammaherpesvirus in a cell line derived from the microbat *Myotis velifer incautus.* This is the first report on a replicating, infectious gammaherpesvirus from bats. The new virus is named bat gammaherpesvirus 8 (BGHV8), also known as *Myotis* gammaherpesvirus 8, and is able to infect multiple cell lines, including those of human origin. Using next-generation sequencing technology, the authors constructed a full-length annotated genomic map of BGHV8. Phylogenetic analysis of several genes from BGHV8 revealed similarity to several mammalian gammaherpesviruses, including Kaposi’s sarcoma-associated herpesvirus (KSHV).

## TEXT

Emerging infectious diseases (EID), a significant financial burden and public health threat, are on the rise (1). The term EID refers to a large range of pathogens from newly drug-resistant microbes to novel viruses (1). Zoonosis is the leading cause of EID (1, 2) and has led to several epidemics in the human population, including the introduction of HIV (3). Bats are recognized as a significant source of zoonosis (4). Severe acute respiratory syndrome (SARS) and Ebola viruses have both been linked to bat reservoirs (5, 6), and both viruses have led to highly infectious and lethal outbreaks in the human population (7, 8). In addition to SARS and Ebola viruses, bats contain and transmit other human pathogens not limited to, but including, Hendra virus (HeV) (9), Nipah virus (NiV) (10), and rabies virus (11, 12). Bats are a source of zoonosis because they embody three factors that allow transmission among themselves and to humans: they have a long life span relative to body size, they live in close colonies, and they are the only flying mammals (13, 14). Upon harboring a potential human pathogen, bats can spread disease through several mechanisms, including saliva, guano, and infection of intermediate hosts (4). Thus, a greater knowledge of the bat viral reservoir and potential of zoonosis may help scientists devise means to prevent future disease outbreaks.

Herpesviruses cause significant human disease. The human gammaherpesviruses Epstein-Barr virus (EBV) and Kaposi’s sarcoma-associated herpesvirus (KSHV) are associated with human malignancies (15–17). The *Gammaherpesvirinae* subfamily is divided into *Macavirus*, *Percavirus*, *Lymphocryptovirus*, and *Rhadinovirus* genera (18). Shabman et al. (19) report the identification of a novel gammaherpesvirus by RNA sequencing (RNA-seq) transcriptomic analysis of an MVI-it bat cell line derived from an interscapular tumor from the microbat *Myotis velifer incautus*. The new virus was named bat gammaherpesvirus 8 (BGHV8) according to the International Committee on Taxonomy of Viruses (ICTV) standards. Transmissibility and infectivity of the novel BGHV8 were established by transferring supernatant from MVI-it cells onto African green monkey (Vero) cells. Vero cell infection was confirmed through microscopic observation of viral cytopathic effect and classical herpesvirus syncytium formation. Further, BGHV8 infection of Vero cells and the human cell lines A549 and Huh7 was confirmed through quantitative PCR (qPCR) verification of viral transcription and BGHV8 genome amplification. Vero cells were used to establish a plaque assay and a 50% tissue culture infectious dose (TCID_50_) assay. Electron microscopy confirmed production of mature viral particles within MVI-it infected cells. Illumina HiSeq was utilized to sequence viral DNA extracted from BGHV8-infected Vero cell supernatant. The HiSeq reads were aligned to construct a genome map of the new virus. Tandem repeats were identified via software prediction tools, and microRNA (miRNA) sequences were identified by small RNA capture methods within the MVI-it cell line. To understand BGHV8’s relatedness to other gammaherpesviruses, the conserved gB glycoprotein was analyzed and found to be similar to, yet distinct from, other gammaherpesviruses. BGHV8 contains several accessory open reading frames (ORFs) homologous to those in KSHV (Table 1), including v-FLIP, E11 (v-OX2), dihydrofolate reductase (DHFR), v-Bcl-2, CCP, and MIR2 ORFs. Gammaherpesviruses are thought to pirate genes from their host, and BGHV8 is similar in this regard, as the v-OX2 viral protein showed homology to the bat cellular OX2 protein (20, 21). Additionally, BGHV8 also contained three unique open reading frames at the 5′ end of the viral genome, which were named U4, U5, and U6. Finally, the mRNA profile of BGHV8 within MVI-it cells showed that the majority of ORFs identified are indeed expressed, with the structural ORFs being among the most highly expressed.

**TABLE 1:** Homology between BGHV8 and KSHV ORFs

BGHV8 ORF^b^	KSHV ORF		
	Name	Function	Amino acid identity/positivity (%)^a^
v-FLIP	v-FLIP	Antiapoptosis	29/51
E11	v-OX2	T-cell attenuation	33/51
DHFR	DHFR	Viral replication	45/62
v-Bcl-2	v-Bcl-2	Antiapoptosis	29/38
sCCPH	KCP	Complement control	28/45
MIR2	MIR2	Immune modulation	29/41

a BLAST protein-protein alignment between BGHV8 KU220026 and human herpesvirus 8 NC_009333 amino acid sequences.

b v-FLIP, viral FADD-like interferon converting enzyme or caspase 8 (FLICE) inhibitory protein; v-OX2, viral OX2; DHFR, dihydrofolate reductase; v-Bcl-2, viral B-cell lymphoma 2; sCCPH, soluble complement control protein homologue; MIR2, modulator of immune recognition 2.

BGHV8 clusters with the *Rhadinovirus* genus, which contains the human pathogen KSHV (22). BGHV8 shares homology with KSHV immunomodulatory genes as depicted in Table 1. The homology between the proteins encoded by BGHV8 and those encoded by KSHV is not very high, and yet it is likely that certain functions are conserved between these proteins, as has been shown previously for KSHV and other herpesviruses (23, 24). Some of the BGHV8 homologues in KSHV play roles in the prevention of apoptosis, immune evasion, and viral replication (Table 1). Additionally, the KSHV v-FLIP protein may also contribute to tumorigenesis (25, 26). Indeed, the MVI-it cell line used in the study was isolated from a bat tumor, suggesting that in concordance with other gammaherpesviruses, BGHV8 may likely also possess oncogenic potential.

Previous reports have shown that multiple gammaherpesviruses can be found in the bat reservoir (27–29); however, this was the first report to identify and characterize a replicating, infectious gammaherpesvirus. Further, betaherpesvirus sequences have been identified within the bat reservoir (27), as well as a replicating betaherpesvirus, suggesting that bats are a suitable host for a range of different herpesviruses (30). As BGHV8 displays some similarity to human herpesviruses, including KSHV, it is somewhat plausible that the human population may have some cross protection against BGHV8. However, there also exists a potential for the transmission of this bat gammaherpesvirus into humans. In summary, the article by Shabman et al. (19) represents an exciting new step in understanding the bat viral reservoir.
